# The utility of flexible and navigable suction access sheath (FANS) in patients undergoing same session flexible ureteroscopy for bilateral renal calculi: a global prospective multicenter analysis by EAU endourology

**DOI:** 10.1007/s00345-025-05477-9

**Published:** 2025-02-28

**Authors:** Vineet Gauhar, Bhaskar Somani, Daniele Castellani, Khi Yung Fong, Nariman Gadzhiev, Satyendra Persaud, Saeed Bin Hamri, Chu Ann Chai, Azimdjon Tursunkulov, Yiloren Tanidir, Boyke Soebhali, Anil Shrestha, Deepak Ragoori, Mohamed Elshazly, Mehmet Ilker Gokce, Vigen Malkhasyan, Yasser Farahat, Thomas Herrmann, Olivier Traxer, Steffi Kar Kei Yuen

**Affiliations:** 1 Department of Urology, Tan Tock Seng Hospital, Singapore, Singapore; 2 Department of Urology, University Hospitals Southampton, NHS Trust, Southampton, UK; 3 Urology Unit, Azienda Ospedaliero-Universitaria delle Marche, Università Politecnica delle Marche, Ancona, Italy; 4 Yong Loo Lin School of Medicine, National University of Singapore, Singapore, Singapore; 5 Department of Urology, Saint-Petersburg State University Hospital, Saint-Petersburg, Russian Federation; 6 Division of Clinical Surgical Sciences, University of the West Indies, St. Augustine, Trinidad and Tobago; 7 Division of Urology, Department of Surgery, Ministry of the National Guard Health Affairs, King Saud Bin Abdulaziz University for Health Sciences, King Abdullah International Medical Research Center, Riyadh, Saudi Arabia; 8 Department of Surgery, Urology Unit, University of Malaya, Kuala Lumpur, Malaysia; 9 Urology Division, AkfaMedline Hospital, Tashkent, Uzbekistan; 10 Department of Urology, Marmara University School of Medicine, Istanbul, Turkey; 11 Department of Urology, Abdul Wahab Sjahranie Hospital Medical Faculty, Muliawarman University, Samarinda, Indonesia; 12 Department of Urology, National Academy of Medical Sciences, Bir Hospital, Kathmandu, Nepal; 13 Department of Urology, Asian Institute of Nephrology & Urology, Irram Manzil Colony, Hyderabad, Telangana India; 14 Urology Unit, Menoufia University, Shibin el Kom, Egypt; 15 Department of Urology, Ankara University School of Medicine, Ankara, Turkey; 16 Endourological Department, A. I. Yevdokimov Moscow State University of Medicine and Dentistry, Moscow, Russian Federation; 17 Endo-Urology & Minimally Invasive Surgery, Sheikh Khalifa Hospital, Dubai, UAE; 18 Department of Urology, Spital Thurgau AG, Kantonsspital Frauenfeld, Pfaffenholzstrasse 4, Frauenfeld, CH 8501 Switzerland; 19 Department of Urology AP-HP, Sorbonne University, Tenon Hospital, Paris, France; 20 S. H. Ho Urology Centre, Department of Surgery, The Chinese University of Hong Kong, Ma Liu Shui, Hong Kong China

**Keywords:** Flexible ureteroscopy, Flexible and navigable suction ureteric access sheaths (FANS), Suction, Urolithiasis, Bilateral RIRS

## Abstract

**Purpose:**

To assess the 30-day stone-free rate and peri-operative outcomes of flexible ureteroscopy (FURS) with flexible and navigable suction ureteral access sheaths (FANS) in adults undergoing same-sitting bilateral retrograde intrarenal surgery (SSB-RIRS).

**Methods:**

Prospectively data of 115 adult patients with bilateral kidney stone disease undergoing SSB-RIRS across 14 global centers between July 2023 and March 2024 were analyzed. Patient demographics, stone characteristics and operative outcomes were recorded. A low-dose non contrast CT scan was performed at 30 days to assess the stone-free rate and clinical outcomes.

**Results:**

Overall bilateral zero residual fragment(ZRF) was 42.6%; unilateral ZRF was 75.7%. Only two patients were noted to have residual fragments > 4 mm. 1.7% experienced Traxer-Thomas grade 1 ureteric injury which was managed with a ureteral stent for four weeks. No pelvicalyceal injury occured. Postoperative mean loin pain score was 1.7 ± 1.0. None had sepsis nor required blood transfusion. 4.3% required readmission within 30 days of surgery. Multivariate analysis indicated longer total operation time correlated with lower odds of achieving a 100% bilateral stone-free (ZRF) (OR 0.978, 95%CI = 0.959–0.994, *p* = 0.013).

**Conclusion:**

To our knowledge, this is the first multicenter study demonstrating the use of FANS in SSB-RIRS can achieve bilateral ZRF with low complication and re-intervention rates. However, prolonged surgical time may negatively impact outcomes. The indications of bilateral renal stones management with FURS can be expanded in appropriate chosen patients.

## Introduction

Bilateral kidney stone disease (KSD) negatively impacts quality of life [1] and doubles the risk of surgical interventions [2]. Clinicians face the dilemma of offering upfront intervention to both sides in a single sitting versus a safer staged approach [3]. The potential advantages of bilateral procedures under a single anaesthesia, such as cost saving and better utilization of theatre resources, make this an attractive option [4, 5]. Evidence showed that bilateral percutaneous nephrolithotomy (PCNL) and bilateral retrograde intra-renal surgery (RIRS) can be safe and effective in adults and children [6–9].

Castellani et al. highlighted that while complication rates are acceptable, the residual fragment (RF) and reintervention rates can be high [8]. A recent randomized controlled trial [10] using the flexible and navigable suction access sheath (FANS) demonstrated significantly improved RIRS outcomes, namely stone-free rates (SFR), infectious complications and re-intervention, making this accessory a potential game changer [11].

This study aimed to assess the 30 day zero residual fragment, single stage overall stone-free rate and report peri-and postoperative outcomes of flexible ureteroscopy (FURS) with FANS in adults undergoing same-sitting bilateral retrograde intrarenal surgery (SSB-RIRS).

## Methods

Adult patients with bilateral KSD who were suitable for SSB-RIRS with FANS were prospectively enrolled into an ethics approved registry (#AINU 28/2023) between July 2023 and March 2024. 115 patients were included across 14 centers, and each surgeon was required to complete a minimum of five cases for successful participation, considering the strict criteria for pre- and post-operative follow-up with non-contrast computer tomography (NCCT) scans.

Inclusion criteria: adult patients with bilateral renal stones in a normal pelvicalyceal system (PCS), in whom FANS successfully deployed bilaterally to perform SSB-RIRS. Patients with concomitant ureteral stones, unable to consent for surgery, anomalous pelvicalyceal anatomy, aged < 18 years, unable to follow up with a low-dose NCCT scan for RF assessment or a follow-up at 30 days post index procedure were also excluded. Pre-operative stone volume (SV) was assessed using the ellipsoid formula [12]. For multiple stones, the stone with largest diameter and volume was considered independently on each side. All positive urine culture were treated as per antibiogram. Pre-stenting was not mandatory, as it has been shown in previous studies that using FANS was possible and safe even in non-stented patients [10, 11].

Baseline patient and stone characteristics, laser and lithotripsy data were gathered. Holmium or Thulium fibre laser were used. The most commonly used FANS models were ClearPetra (Well Lead Medical, Guangzhou, China) and Innovex Medical (Shanghai, China). The ease of use, manoeuvrability within PCS and the bilateral utility of FANS as a whole was documented by a 5-point Likert-type scale (score:1 excellent; 5: difficult) [13]. Total operative time was defined as the time from start of cystoscopy to exit strategy (stent, ureteric catheter, or nil drainage) after completion of both sides. Total ureteroscopy time was the time from FANS placement to its removal after using bilaterally. Total Laser time was obtained from the machine display. Other variables included complications within 24 h, such as bleeding requiring transfusion, ureteric injury, PCS injury, infective complications, and loin pain score using a 10-point visual analog scale [14] (1 being the lowest score). SFR and all-cause readmission within 30 days and future planned re-interventions were documented. All surgeons were instructed to visually inspect each side and perform a retrograde pyelogram to ensure the integrity and condition of the ureteral mucosa and to clearly document any injury according to the Traxer-Thomas classification [15].

### Outcomes

SFR and RF were assessed by the bone window of 4weeks post-index procedure NCCT scan. **Each patient** would fall under at least one category:

- Grade A(100% stone-free): no RF/ ZRF bilaterally.
- Grade B: single RF < 2 mm in maximum diameter at least unilaterally.
- Grade C: single RF 2.1–4 mm in maximum diameter unilateral or bilaterally.
- Grade D: single or multiple RF > 4 mm in maximum diameter unilateral or bilaterally.

As per EAU guidelines [12], significant RF burden, often defined as fragments 3–4 mm or more, may need intervention or follow up with active surveillance as to mitigate significant clinical events or progression.

- Bilateral Grade A(100% SFR): no intervention or follow up is needed.
- Grade B and C are insignificant RF burden and do not warrant surgical intervention.
- Grade D RF may need surgical re-intervention or active surveillance.

### Statistical analysis

Continuous variables are expressed as medians and interquartile ranges while categorical variables are reported as absolute numbers and percentages. Multivariable logistic regression analysis was utilized to derive significant predictors for the outcome of 100% SFR(bilateral Grade A). Predictors were expressed as odds ratios(ORs) and 95%CI. Statistical analyses were performed using R-4.3.0(R Foundation for Statistical Computing, Vienna, Austria) with *p* < 0.05 indicating statistical significance.

## Results

### Patient demographics and stone features (Table 1)

**Table 1:** Baseline characteristics. Expressed as median [interquartile range] for continuous variables and n (%) for categorical variables

	Overall (*N* = 115)	
Age	44 [34–58]	
Male gender	59 (51.3)	
ASA grade		
1	65 (56.5)	
2	44 (38.3)	
3	6 (5.2)	
BMI, kg/m^2^	26 [24–30]	
Anticoagulants/antiplatelets (stopped 5 days before op)	28 (24.3)	
First-time stone formers	73 (63.5)	
First clinical presentation		
Haematuria	9 (7.8)	
Pain	66 (57.4)	
Fever	13 (11.3)	
Incidental	41 (35.7)	
Positive urine culture (treated with preoperative antibiotics)	24 (20.9)	
Preoperative antibiotics (therapeutic/ or prophylactic)	111 (96.5)	
Prestented (unilaterally or bilaterally)	79 (68.7)	
	Left side	Right side
Stone diameter		
< 1 cm	49 (42.6)	61 (53.0)
1.1–2 cm	56 (48.7)	47 (40.9)
> 2 cm	10 (8.7)	7 (6.1)
Stone volume, mm^3^	1407 [840–2650]	1342 [795–2285]
Stone Hounsfield units	1012 [890–1237]	1020 [850–1200]
Stone location		
Upper pole	15 (13.0)	10 (8.7)
Interpolar/pelvis	35 (30.4)	32 (27.8)
Lower pole	25 (21.7)	26 (22.6)
Multiple locations	40 (34.8)	47 (40.9)

ASA – American Society of Anesthesiologists; BMI – body mass index

115 patients were analysed. Gender distribution remained balanced. 63.5% were first time stone formers, presented primarily with pain on at least one side. 68.7% had been pre-stented for symptomatic relief or pre-emptively. Stone diameters were categorized into < 1 cm, 1.1–2 cm, >2 cm, of which the distribution of stone diameters was 42.6%, 48.7%, 8.7% respectively on the left side; and 53.0%, 40.9%, 6.1% respectively on the right side. Median stone volumes were 1407 mm^3^(left) and 1342 mm^3^(right). Median Hounsfield units were 1012(left) and 1020(right).

### Perioperative characteristics and outcomes (Table 2)

**Table 2:** Operative characteristics, postoperative complications and 30 day stone-free rates. Expressed as median [interquartile range] for continuous variables and n (%) for categorical variables

	Overall (*N* = 115)		
Thulium fibre laser (TFL)	84 (73.0)		
Total operation time (Bilateral), min	70 [60–92]		
Ureteroscopy time (Bilateral), min	56 [44–80]		
Laser time (Bilateral), min	16 [12–23]		
Scope size, Fr			
7.5	54 (47.0)		
8.5	14 (12.2)		
8.6	20 (17.4)		
9.5	27 (23.5)		
Need for basketing (for fragments extraction or repositioning)	38 (33.0)		
UAS change due to malfunction	2 (1.7)		
Sheath size			
12–14	40 (35.4)		
11–13	33 (28.6)		
10–12	42 (37.2)		
Sheath able to access whole kidney	28 (25.0)		
Successful sheath access to bilateral lower pole	87 (77.0)		
Suction worked well	113 (98.2)		
Suction failure due to machine issues	2(1.7)		
Intraoperative SFR assessment			
Bilateral 100% SFR	28 (24.3)		
Dust only, no fragments	67 (58.3)		
Fragment(s) on either side	20 (17.4)		
Exit strategy			
Bilateral stent	87 (75.7)		
Unilateral stent + overnight Indwelling ureteric catheter	2 (1.7)		
Unilateral stent only	7 (6.1)		
Bilateral overnight indwelling ureteric catheter only	14 (12.2)		
No stent and no indwelling ureteric catheter bilaterally	5 (4.3)		
Likert scale rating for bilateral utility of FANS (1 excellent, 2 very good, 3 good, 4 average, 5 poor)			
Ease of using suction	1(1–2)		
Manoeuvrability into calyces	1(1–2)		
Contributing to visibility	1(1–2)		
	Left side	Right side	
Fragmentation modality			
Dusting + suction	22 (19.1)	20 (17.4)	
Popcorning + suction	9 (7.8)	3 (2.6)	
Fragmentation + suction	8 (7.0)	10 (8.7)	
Combination	76 (66.1)	82 (71.3)	
Stone freedom parameters on CT scan			
100% SFR bilateral (Grade A) Bilateral Zero residual Fragment (ZRF)	49 (42.6)		
100% SFR in any or both sides (Grade A) At least unilateral zero residual fragment (ZRF)	87 (75.7)		
Any RF Grade C	14 (12.1%)		
Any RF Grade D	2 (1.7%)		
Decision to reintervene	3 (2.6)		
Duration of hospital stay, days	2 [1–3]		
Intraoperative complications			
Bleeding due to sheath movement but not needing intervention	21 (18.3)		
Ureteric injury managed by stent placement (CD2)	2 (1.7)		
Pelvicalyceal system injury (CD2)	0		
Postoperative complications			
Fever within 24 h (CD1)	19 (16.5)		
Fever prolonging stay ≥ 48 h (CD1)	4 (3.5)		
Sepsis (CD4)	0		
Haematuria requiring transfusion (CD2)	0		
Persistent loin pain prolonging admission	0		
Loin pain score (Likert scale analysis)	1 [1–2]		
AVM requiring embolization	0		
Readmission	5 (4.3)		
Reason for readmission (UTI/Stent symptoms/fever/sepsis)	2/2/1/0		

SFR – stone-free rate; FANS – flexible and navigable suction ureteral access sheaths; RF – residual fragment; CD – Clavien-Dindo; AVM – arteriovenous malformation; UTI – urinary tract infection

Median operative time, ureteroscopy time, and laser time were 70, 56 and 16 minutes respectively. 7.5Fr scopes used in 47% and 10/12Fr FANS in 37.2%. Thulium Fiber Laser (TFL) was utilized by 73%. Suction worked in 98.2% cases and in 77% of the cohort, access to lower pole of bilateral kidneys was successfully achieved. Surgeons highly rated the utility of FANS (Likert score 1, average:1–2) for ease of suction, ability to manoeuvre the sheath to various calyces during suction and effectively contributing to vision while performing laser lithotripsy. 33% required stone baskets for fragment extraction or repositioning. Only two cases (1.7%) required a change to a new ureteral access sheath. In one case, the change was due to a smaller sheath size, while in the second case, the reason was unspecified.” As exit strategy, most (75.7%) had bilateral ureteral stents placed, with 12.2% having bilateral ureteric catheters removed the following morning.

Overall bilateral 100% SFR (i.e. bilateral ZRF) in 42.6% and unilateral Grade A(ZRF) in 75.7% cases. Only 2 (1.7%) patients noted to have RF > 4 mm in size(Grade D). A repeat FURS intervention was proposed in just 3(2.6%) patients. 1.7% experienced a Traxer grade 1 [15] ureteric injury managed with a ureteral stent for 4 weeks. None reported pelvicalyceal injury. Postoperative mean loin pain score was 1.7 ± 1.0. 16.5% had fever on POD 1 (> 38.5) that responded within 24 h. None had sepsis nor required blood transfusion. 4.3% required readmission within 30 days of surgery, 2 for urinary tract infection, 2 for stent related symptoms and 1 for low grade fever.

Multivariate analysis (Table 3) indicated longer total operation time correlated with lower odds of achieving a 100% bilateral SFR(Grade A) (OR 0.978, 95%CI = 0.959–0.994, *p* = 0.013).

**Table 3:** Multivariable analysis of 100% SFR (zero residual fragment bilaterally) on postoperative CT scan

	HR	95%CI	*p*-value
Total stone volume	1	1.000–1.000	0.191
Thulium fiber laser (TFL)	0.614	0.192–1.958	0.406
Sheath size (vs. 12–14)			
11–13	2.671	0.701–11.097	0.159
10–12	0.796	0.166–3.742	0.771
Scope size, Fr (vs. 7.5)			
8.5	0.674	0.119–3.797	0.652
8.6	0.8	0.150–3.934	0.787
9.5	2.903	0.644–13.903	0.169
Total operation time (both sides), min	0.978	0.959–0.994	**0.013**

HR – hazard ratio; CI – confidence interval

## Discussion

As surgeons embrace smaller diameter scopes, access sheaths and better lasers, same sitting bilateral endourological surgeries have gained momentum despite the lack of definitive guidelines. Real world study of 1250 patients by Castellani et al. [8] showed that bilateral FURS can be effective with 73% bilateral SFR and 37% needing reintervention at 3 months. The high reintervention rate is counterproductive if the main reason to attain a high SFR and maximise utility of operative resources in single intervention is not achievable.

In our study, only bilaterally successfully performed cases were included, while those in which FURS failed were excluded. Although guidelines do not mandate pre-stenting [12], FANS has been performed in non-stented patients [10, 11], 68.7% of patients in our series were pre-stented unilaterally or bilaterally (Table 1). Studies have shown that pre-stenting facilitates UAS placement and minimizes access sheath injury [16, 17]. This may explain why, in 115 patients, FANS-UAS was successfully placed bilaterally with low ureteric injury rate of 1.7%. While we cannot advocate pre-stenting as the standard of care, we acknowledge that it is an issue that urologists should discuss with patients to improve access success and ensure safe operation. Potentially, pre-stenting allows passive ureteral dilation which may facilitate pelvicalyceal manipulation, especially when addressing lower pole stones [18, 19].

In this first report of SSB-RIRS, we were able to achieve bilateral ZRF in 42.6% and unilateral ZRF in 75.7%. At 30 days, only 2(1.7%) patients had RF > 4 mm, 2.65% had planned re-intervention. 16.5% of patients experienced fever on postoperative day 1 (> 38.5 °C), which responded within 24 h to a single dose of antibiotics. Only four patients had a fever that persisted beyond 48 h. Fever after endoscopic intervention is multifactorial [20, 21]. Multiple factors, including larger stone volume, positive preoperative urine culture, multiple stones, indwelling preoperative stents, use of ureteric access sheath (Tables 1 and 2, and 3), and longer surgical time, likely account for the increased incidence of fever in our study. While this incidence is higher than that reported in unilateral FANS [10], it is comparable to that seen in series of flexible ureteroscopy for bilateral stones [5, 8]. Notably, our series reported a zero sepsis rate with low pain score, demonstrating that SSB-RIRS with FANS is safe and may potentially change approach in bilateral KSD.

In this global real world data, there is an average operative time of just 78 min, well within the 90 min safe operative time proposed by studies [11]. Indeed, the same FANS sheath was able to successfully access the lower pole of both kidneys in 77% of cases. While unilateral access to the lower pole could have been higher, this information was not gathered and thus not interpreted. At the same time, surgeons used baskets for fragment repositioning or removal in 33% of cases. This rate is significantly higher compared to 13.2% in the global FANS series [11], but closer to the 28.1% reported by Zhu et al. [10]. However, these figures are much lower than those reported in the SSB-RIRS series by Castellani D et al. [8], where 45.3% of patients required baskets, where the overall bilateral SFR was only 73%.

While it was not the primary aim of this study, it has been postulated that as technology improves and we gain experience with FANS and suction modalities, the need for baskets may decrease. Until then, surgeons should not hesitate to use them as needed. In a recent study by Kwok et al. [22], smaller sheath sizes demonstrated better access to all parts of the kidney, including the lower pole, and had a higher chance of achieving a zero fragment rate (68% vs. 53%, *p* = 0.02). Similarly, in a study by Lua A et al., intra- and inter-scope comparisons of maximum deflection angles of various sheaths were significantly different when used with different scopes [23]. This is an important consideration when using different scopes and sheath sizes. Furthermore, they advocate that the sheath advancement technique is significantly better for accessing the lower pole compared to active deflection of the tip.

While we do not have such data collected for this study as these are relatively new, our analysis revealed that sheath size did not show any significant impact on stone-free status. This is perhaps something to consider for future studies, which may further improve the outcomes of bilateral FANS and reduce the need for basket repositioning.

Our results albeit bilateral echoed with those from the recent randomized controlled trial by Zhu et al. [10] that demonstrated unilateral RIRS with FANS has high immediate SFR compared to conventional UAS group (81.3% vs. 49.4%) and at 3 months (87.5% versus 70.0%). We too had low fever rates, no sepsis, reduced use of stone baskets and excellent pain scores with short hospital stay, making this instrument a potential game changer in SSB-RIRS. As these results were conclusively reported with a NCCT within 30 days with very low perioperative complications, and a low rate of ureteric injury despite deploying FANS bilaterally, it reflects that in normal kidneys FANS may be used safely. FANS improves the quality of SSB-RIRS as dust, debris and fragments are aspirated simultaneously with laser lithotripsy. Importantly, as has been explained in other studies using suction lowers intrarenal pressure (IRP), the salient reason for mitigation septic complications and reducing post operative pain caused by overstretching of renal capsule [11, 24–27]. In our study, we also included patients with stones larger than 2 cm (8.7% on the left side, 6.1% on the right side). The successful intervention of these larger stones using FANS in a single-stage procedure underscores the advantages of suction aspiration for managing large stone burdens, as highlighted in other studies [28]. PCNL remains a valid option for such stone burden [12]. Although it is beyond the scope of our study, a comparison of simultaneous bilateral PCNL versus bilateral RIRS utilizing FANS would be a valuable area for future research.

### Study limitations and future directions

Traditionally, surgeons have approached bilateral KSD by treating only the symptomatic side while either observing the asymptomatic side or planning a staged surgery [3, 29, 30]. With current advancements, surgeons may reconsider strategies, expanding the indications for performing bilateral RIRS in single sitting intervention.

Our multivariable analysis infers that the key to achieving bilateral ZRF was appropriately chosen stone volume to minimise the operative time. Stratification by stone diameter is the most common method for planning RIRS, as outlined in the EAU guidelines [12]. Nonetheless, a bilateral procedure, where many permutations and combinations of stone size, location, and multiplicity are expected, all add to the complexity of the procedure. Surgeons should be cautious about relying on stone diameter, as stone volume is a more accurate predictor of stone burden [31]. We reported the largest stone volume rather than the cumulative stone volume, which would better reflect the total stone burden the surgeon must manage in a single sitting. Our outcomes can serve as a reference guide to better counsel patients considering SSB-RIRS with FANS, encouraging future research into cumulative stone volume measurements.

The high proportion of pre-stenting in our series raises questions about whether this was a matter of preference or a clinical necessity. It is crucial to discuss the pros and cons of pre-stenting with patients, weighing on-table success against potential extra procedural costs and risks.

In our study, FANS in SSB-RIRS resulted in low RF achieved by thorough navigation of the sheath into all calyces to visually confirm stone clearance [11, 24, 27]. This led to a low reintervention rate (2.6%), lower than the 9.9% reported in the literature, thereby reducing the need for secondary procedures, translating to fewer hospital visits and lower healthcare costs.

A practical consideration is the ergonomics of performing bilateral RIRS [32]. Whilst not assessed, suction aspiration of fragments though the FANS sheath involves multiple withdrawals of the scope all the way into the distal part of the sheath till the Y connector and can potentially cause wrist strain. More information on laser lithotripsy strategy to improve efficacy is needed.

Despite preoperative antibiotic treatment and the use of suction measures to minimise post FURS infective complications, a incidence of fever in 16.5% within 24 h remains a critical consideration for centres which practice RIRS as an ambulatory surgery [3, 5, 6, 8, 11].

In summary, our data address concerns that have historically deterred urologists from offering bilateral surgeries, supporting increased adoption of SSB-RIRS with FANS. The strengths of this study include its prospective multicenter design, large sample size, and thorough reporting of relevant outcomes. However, the absence of a control group limits the ability to attribute observed benefits solely to the use of FANS. Comparative studies [10, 33] have indicated that FANS significantly outperforms non-suction ureteral access sheaths across various operative parameters, potentially establishing a baseline for future bilateral RIRS using suction modalities, which promise to be transformative in endourology [34, 35].

## Conclusion

To our knowledge, this is the first multicenter study where the use of FANS in SSB-RIRS can achieve bilateral zero residual fragments with low complication, zero sepsis and low reintervention rates. Same siting bilateral RIRS with FANS is a safe modality for bilateral renal stone if used carefully. As we learn how to better use FANS in RIRS, these findings if replicated could change the way urologists’ approach in bilateral renal stone management.

## Data Availability

No datasets were generated or analysed during the current study.
